# An Agent-Based Modeling Template for a Cohort of Veterans with Diabetic Retinopathy

**DOI:** 10.1371/journal.pone.0066812

**Published:** 2013-06-21

**Authors:** Theodore Eugene Day, Nathan Ravi, Hong Xian, Ann Brugh

**Affiliations:** 1 Health Services Research and Development, VA St. Louis Healthcare System, St. Louis, Missouri, United States of America; 2 Ophthalmologist, VA St. Louis Healthcare System, St. Louis, Missouri, United States of America; 3 Department of Ophthalmology and Visual Sciences, Washington University in St. Louis, St. Louis, Missouri, United States of America; 4 Department of Energy, Environment and Chemical Engineering, Washington University in St. Louis, St. Louis, Missouri, United States of America; 5 Department of Biostatistics, St. Louis University, St. Louis, Missouri, United States of America; 6 Primary Care Mental Health Integration, VA St. Louis Healthcare System, St. Louis, Missouri, United States of America; University of Tennessee, United States of America

## Abstract

**Background:**

Agent-based models are valuable for examining systems where large numbers of discrete individuals interact with each other, or with some environment. Diabetic Veterans seeking eye care at a Veterans Administration hospital represent one such cohort.

**Objective:**

The objective of this study was to develop an agent-based template to be used as a model for a patient with diabetic retinopathy (DR). This template may be replicated arbitrarily many times in order to generate a large cohort which is representative of a real-world population, upon which *in-silico* experimentation may be conducted.

**Methods:**

Agent-based template development was performed in java-based computer simulation suite AnyLogic Professional 6.6. The model was informed by medical data abstracted from 535 patient records representing a retrospective cohort of current patients of the VA St. Louis Healthcare System Eye clinic. Logistic regression was performed to determine the predictors associated with advancing stages of DR. Predicted probabilities obtained from logistic regression were used to generate the stage of DR in the simulated cohort.

**Results:**

The simulated cohort of DR patients exhibited no significant deviation from the test population of real-world patients in proportion of stage of DR, duration of diabetes mellitus (DM), or the other abstracted predictors. Simulated patients after 10 years were significantly more likely to exhibit proliferative DR (P<0.001).

**Conclusions:**

Agent-based modeling is an emerging platform, capable of simulating large cohorts of individuals based on manageable data abstraction efforts. The modeling method described may be useful in simulating many different conditions where course of disease is described in categorical stages.

## Introduction

### VA St. Louis Healthcare System

The Veterans Administration (VA) St. Louis Healthcare System is a tertiary care facility with 218 medical and surgical beds, serving more than 55,000 Veterans in eastern Missouri and southern Illinois. The eye clinic is staffed by three attending, two resident and two student optometrists, as well as 3.8 full time-equivalent ophthalmologists. The clinic sees approximately 14,000 unique patients annually, including 955 patients with a primary diagnosis of diabetic retinopathy as of 2011. Current VA policy is that all diabetic patients receive a diabetic eye exam at least once every year. This suggests that some 3,500 diabetic eye exams will need to be performed annually, given that the rate of diabetes in the Veteran population is more than 25% [1].

### Diabetic Retinopathy

Diabetes is epidemic in the United States and growing, with nearly 1 in 10 adults over the age of 45 suffering from the disease [2]. According to Miller et al., annual expenditures on diabetes treatment and care are nearly $100 billion, and consume nearly 15% of all health care outlays. In the Veteran population, diabetes is far more prevalent than the population as a whole, even restricted to adults, with greater than 25% of Veterans suffering [1]. Of the population of adults with diabetes, 28.5% suffer from diabetic retinopathy (DR), and 4.4% from vision threatening DR, costing over $500 million dollars annually [3]. DR is the leading cause of new blindness in adults 20–74 [4].

### Agent-based Modeling

Agent-based models (ABM) are formulated around the creation of a cohort of objects, known as “agents”, which are placed in an environment and imbued with individuated attributes and rules for interaction with the environment and with other agents. These agents represent individual members of a larger group. They may be seen as individuals within a population, or as cells in an organism. Use of computer simulation for the study of complex systems employing agents is a relatively new endeavor, emerging during the mid 1990’s [5]. These systems are characterized as consisting of interactive autonomous agents, the environment they inhabit, and by exhibiting - in the words of Forrest and Jones - “[u]nanticipated global properties”. (This is generally referred to today as “emergent behavior”.) As dynamic models, agent-based models evolve over time, and are designed to simulate real-world complex systems which do the same. ABMs allow us to examine population level effects of diffusion of information and adoption of behaviors [6].

ABM is being used effectively in a number of capacities in medical research. The early uses of ABM in medicine were at the level of biological systems, for example modeling inflammatory response [7], granuloma formation in tuberculosis [8], and immune response [9]. More recently, the potential for ABM to provide relevant and enlightening results in the field of epidemiology has begun to be realized. The capacity for agents to represent human individuals, imbued with a demography representative of a real-world population opens the potential for *in-silico* epidemiological studies [10]. ABM also has demonstrated application to the field of human behavior study [11]–[12], and in more traditional settings of scheduling in medical environments [13]. Additionally, ABM has been shown to provide value in predicting ophthalmic outcomes as well, particularly in the field of age-related macular degeneration [14]–[15].

### Objective

This paper describes the development of a template for a diabetic agent, in the context of an agent-based model of a cohort of patients with or at risk for DR. Our goal is to describe the process of the creation of an agent, the process of informing that agent with data abstracted from medical records, and the replication of a simulated cohort of identical size to the retrospective, real-world cohort. We additionally present the methodology for validating the simulated cohort against real-world test data. Finally, we allow the cohort to age ten simulated years, and report the outcomes.

## Methods

### Cohort Selection and Data Abstraction

The population of real-world patients constituting the cohort for this study will be those patients who have sought surveillance or treatment for DR at the VA St. Louis Healthcare System eye clinic during the period from CY 2006–2010. We restrict to those patients determined to be definitely diabetic, as defined by the ProClarity Diabetes Cube: either one inpatient or two outpatient diagnoses, or a prescription for a greater than 30 day supply of diabetes specific pharmaceuticals. We further restrict to those patients with a primary diagnosis of one of four ICD9 codes for ophthalmic complications: 362.0 for proliferative, non-proliferative, and background diabetic retinopathy or 362.10 background retinopathy unspecified, 379.23 vitreous hemorrhage, and 250.5 diabetes mellitus with ophthalmic manifestation. This allows us to identify only those patients exhibiting non-proliferative diabetic retinopathy (NPDR), and proliferative diabetic retinopathy (PDR).

This resulted in 535 records patient records. Elements abstracted included patient age, gender, BMI, current HbA1c (glycated hemoglobin); dichotomous variables included hypertension, dyslipidemia, diabetic nephropathy, and current tobacco use. Finally, current state of DR (mild, moderate or severe NPDR, or PDR) was abstracted. Of the identified records, duration of diabetes could not be determined in 34 patients, and the records were excluded. Finally, the remaining set of 501 records was randomly divided into the training (n = 351) and test (n = 150) data sets. The sample sizes were chosen to have as large a training set as possible while maintaining >90% statistical power to detect a 10% variation in the continuously distributed predictors in the test data set. This protocol was reviewed and approved by the VA St. Louis Healthcare System Institutional Review Board, and a Waiver of Informed Consent was granted for collection of existing data.

### The Statechart Model

**Figure 1:**
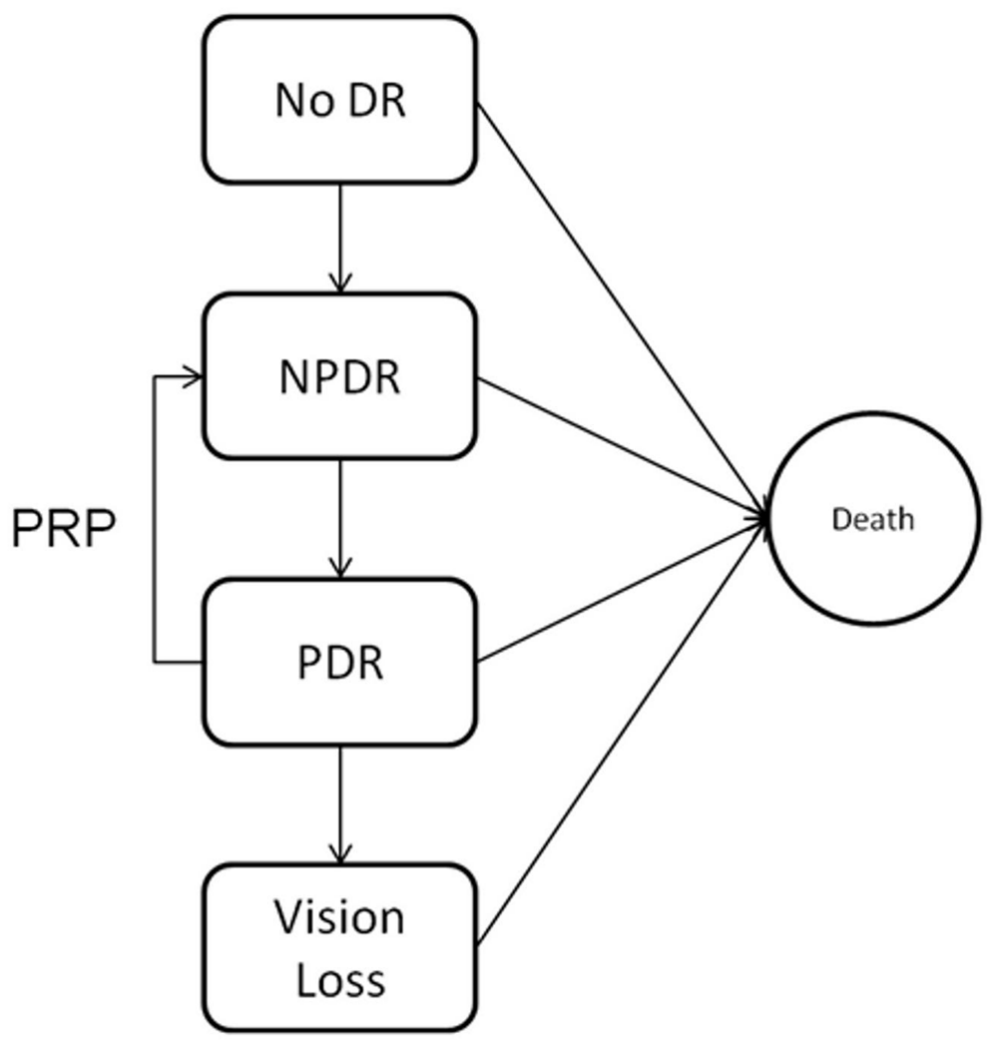
Statechart model for Diabetic Retinopathy. DR: Diabetic Retinopathy NPDR: Non-proliferative Diabetic Retinopathy PDR: Proliferative Diabetic Retinopathy PRP: Pan-retinal Photocoagulation.

The Agent template was designed using AnyLogic Professional 6.6, a Java-based simulation suite capable of multiple types of simulation modeling. The fundamental structure used to model the progression of DR within the agent is the ‘statechart’. At the most basic conceptual level, a statechart is simply a discretized condition, and rules for how that condition changes. In practice, a statechart is a graphically represented collection of ‘states’, conditions which an agent may inhabit, and ‘transitions’, rules for how an agent exits one state and enters another. Statecharts are easily represented as directed graphs, as the statechart for DR shown in figure 1, where states are nodes on the graph (generally enlarged for clarity), and transitions are edges, with arrowheads showing their direction of action.

The statechart consists of five possible states: (1) no retinopathy, (2) non-proliferative diabetic retinopathy (NPDR), (3) proliferative diabetic retinopathy (PDR), (4)legal vision loss (VL), and (5) a final state called ‘death’. Because the agent model was developed to be used to simulate the course of disease in a dynamic population over the course of many years, agent capacity to exit the cohort was included in the model. Agent death is modeled according to life tables published by the Social Security Statistical Tables website (www.ssa.gov/OACT/STATS/). The initial population of agents is designed to mimic the population of Veterans at VA St. Louis Healthcare System who already carry a diagnosis of DR in some form. Therefore, for validation purposes, we defined the state “no retinopathy” to be uninhabited at model initiation. However when new agents enter the cohort during the simulation run, they are initially assigned to inhabit state (1) with p = 0.715 [3].

### Additional Fields Associated with the Agent

In addition to the statechart, each agent is imbued with several data structures necessary to simulate the progression of DR. First, there are variables, or fields, describing the demography and health status of the agent. These are age, gender, duration of diabetes, BMI, recent HbA1c, and four dichotomous variables indicating presence or absence of hypertension, dyslipidemia, diabetic nephropathy, and current tobacco use. These represent the predictors *a priori* hypothesized to be associated with the progression of DR, or necessary to thoroughly characterize the population simulated. Distribution of values for these fields for the training and test data sets may be seen in table 1. The abstracted data were fit to probability density functions using Stat::Fit Version 2 (Geer Mountain Software Corporation, South Kent, Connecticut), which were called to inform the individual agents upon initiation of the simulated cohort.

**Table 1:** Training, Test, and Simulated Data.

	Training Data	Test Data	Simulated Data	Test Type	P value
N	351	150	501		Test data to Simulated data
Age -yr	67.5±9.4	67.3±8.9	66.6±8.7	t test	0.382
Gender- male	345 (98.3)	148 (98.7)	494 (98.6)	? square	0.476
Duration of DM - yr	21.5±9.0	20.5±8.6	21.1±8.8	t test	0.451
BMI	32.2±6.3	32.3±6.8	31.9±6.6	t test	0.537
A1c	8.2±1.8	8.0±1.5	8.2±1.7	t test	0.359
Hypertension	344 (98.0)	149 (99.3)	488 (97.4)	? square	0.153
Dyslipidemia	297 (84.3)	137 (92)	439 (87.6)	? square	0.139
Diabetic Nephropathy	124 (35.3)	51 (34)	186 (37.1)	? square	0.485
Current Tobbaco User	88 (25.1)	25 (16.7)	110 (21.9)	? square	0.161
NPDR	258 (73.5)	121 (80.7)	369 (73.6)	? square	0.081
PDR	93 (26.5)	29 (19.3)	132 (26.4)		

DM – Diabetes Mellitus.

NPDR - non proliferative diabetic retinopathy.

PDR - proliferative diabetic retinopathy.

BMI - body mass index.

A1c = Hemoglobin A1c.

Values reported as average ± standard deviation, or number (percent).

P value represents comparison of Test Data to Simulated Data.

The final data structure included as part of the agent is a subroutine of computer code called an ‘event’. This event functions as an annual update for the agent, describing the change in the predictors from year to year. The event increases both age and duration of DM by one, and determines whether initial onset of DR, or initial onset of vision loss, occurred during the past year.

### Calculating Probability of DR Progression

The multivariate logistic regression model provides a function, which may be expressed as a probability in the form:
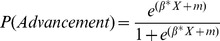
where **β** and m represent the coefficient vector and intercept, respectively, from the logistical regression, and **X** represents the vector of values of the predictors themselves. The annual ‘event’ generates these probabilities based on the agent’s own unique predictors, and then generates a uniformly distributed random number on [0,1] to test if the probability of advancement is satisfied during that year. If appropriate, the agent updates its individualized state-chart, transitioning from NPDR to PDR.

There are four other types of transitions: the first is the transition from ‘no-retinopathy’ (state 1) to ‘NPDR’ (state 2). Because the real-world cohort was restricted to patients already exhibiting DR, the expected time from diagnosis of DM to onset of mild non-proliferative retinopathy was determined from local administrative data, showing a 7.7% annual incidence. This is in line with estimates from the literature, with various reports showing incidence rates of 8.0%–8.4% in recent Australian populations [16], 3.9%–7.1% in a population of Medicaid patients from the 1990s [17]. The second is for transition from proliferative retinopathy (state 3) to vision loss (state 4)for which rates were taken to be 0.7%, annualized from published incidences [18]. The probability of transition from any state to the state ‘death’ (state 5) is computed by accessing the life table described above, using the agent’s age as an input. Lastly, agents are allowed to transition from PDR back to NPDR through the application of pan-retinal photocoagulation, which may be scheduled after the transition from NPDR to PDR.

## Results

### Predictors Associated with DR Progression

Odds ratios were computed using SAS software (SAS 9.2, Cary, NC, USA). Three of the abstracted predictors were found to have statistically significant associations with DR progression: duration of DM (OR 1.059, 95% CI 1.033–1.086), presence of diabetic nephropathy (OR 2.163, 95% CI 1.398–3.346), and BMI (OR 0.961, 95% CI 0.927–0.996). The counterintuitive role of BMI may be due to the truncated nature of the data, which included very few normal- or under-weight individuals. Age, gender, hypertension, dyslipidemia, current tobacco use, and recent HbA1c were all found to lack statistical significance with regard to stage of DR in our cohort.

### Validation of Simulated Cohort against a Real-World Test Cohort

We generated a simulated cohort of 501 individuals, in order to replicate the real-world cohort size. The initial simulated cohort did not show any significant variation from the test cohort in any measured value. Student’s t-tests were used for age, duration of DM, BMI, and HbA1c. χ^2^ tests were used for the dichotomous values of hypertension, dyslipidemia, diabetic nephropathy, tobacco use, and initial stage of DR, either NPDR or PDR. As is necessary for all simulation validation, the ‘test’ data set were not used in training the simulation or generating distributions of predictors. Therefore, comparing the simulated cohort to the test cohort demonstrates that we are capable of generating a population of agents which accurately characterizes the population of Veterans served by VA St. Louis Healthcare System, rather than duplicating a specific subset of those individuals. Complete distributions, specific statistical tests, and p-values are shown in table 1. It is important to note that in contrast to most commonly reported p-values, for purposes of cohort validation, we desire non-significance (i.e., p>0.05); that is, we seek to show that there is no statistically significant difference between the two populations in the tested parameter.

### Demographics of Simulated Cohort after 10 Simulated Years

**Figure 2:**
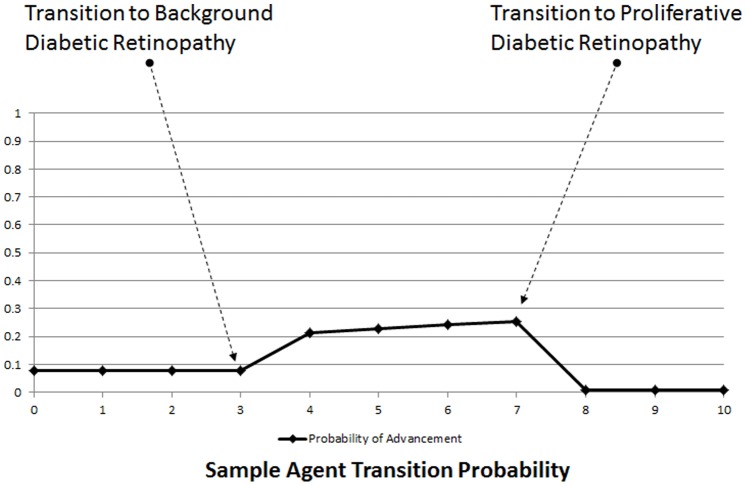
Probability of Agent Advancement. The sample agent’s probability of DR progression over time.

The model was then run forward in time 10 years, and the population examined again. Of the 501 agents beginning the simulation, 327 survived to its termination. The agents which entered state 5 (death) during the simulation were older, (78.1 vs. 74.9, p<0.0001), but had shorter duration of DM (26.4 vs. 30.4, p<0.0001). This is as we would expect: agents beginning the simulation with a lesser age were less likely to die during the ten year period. Thus, they had more time to accrue years of duration of their diabetes. Surviving patients were overwhelmingly in DR state 2, PDR, with 244 inhabiting this state, 78 with NPDR, and 5 with vision loss. Patients not surviving to the end of the simulation were more evenly split, with 98 cases of NPDR, and 76 cases of PDR (p<0.0001). A sample agent’s probability of advancement, prepared separately, is shown in Figure 2. When initialized without DR, the agent has a 7.7% probability of developing incident BDR. After 3 years, BDR develops, and the probability of developing PDR is based on the multivariate regression described above. After year 7 the agent develops PDR, and the transition probability drops to 0.7% annual incidence of vision loss. The sample agent shown does not develop VL during the ten-year simulation period, and does not die during the ten-year simulated period.

## Discussion

Health services research, in the VA and elsewhere, is increasingly focused on implementation science and policy research. As we consider the extraordinary demand the health care system is expected to confront over the coming decades, we must develop tools that will allow us to identify those interventions and policies most likely to have large positive effects on our ability to provide care for a growing and aging population. Good health care policy must first be informed by what it is possible to achieve. Determining the maximum effect sizes of various interventions and policies, in terms of their ability to influence access to care or patient outcomes, will be critical in determining the overall value of potential policies. The development of tools which can rate potential interventions according to their likely impact on patient outcomes and access will provide important evidence either recommending or discouraging adoption, where previously such evidence was subject to large error as a result of speculation.

Agent-based modeling is exactly one such tool. The development of simulated cohorts of agents, which mimic real-world populations of patients, allows us to test *in-silico* interventions which previously could not be tested at all prior to adoption, or which would be prohibitively expensive or harmful in trials should they turn out to be ineffective. While the state of the art of agent-based models is insufficient to, for example, replace real-world clinical trials, the current work shows that they are capable of simulating very realistic cohorts of patients. These cohorts may then be examined under various test conditions, with population-level outcomes used as hypothesis testing criteria.

The development of the simulated cohort essentially creates a sandbox, in which various policy, capacity, and implementation concepts may be tested for efficacy and efficiency. Having this cohort available for such purposes is beneficial in at least three ways. First, we are able to test ideas without making large sunk-cost investments with uncertain outcomes. Second, we are able to experiment with a realistic population while shielding patients from any risk associated with planned interventions. Third, by allowing us to determine the likely limits of an intervention’s effect size, agent-based models show potential for the development of evidence-based performance measures. Evidence-based performance measures will allow us to take into account not only the patient population and best-practices recommendations for care, but also the systems-level evidence of what the delivery apparatus, that is, the clinical and hospital systems, can provide.

These models may also be employed in health economics research. Because they allow us to generate cost functions at the individual patient level, we can calculate expected costs of various policies, interventions, and implementations in response to complex scenarios. Their sensitivity is greater than large scale probabilistic models, as they can predict interactions among the agents (and, if modeled, their environment), which may be unpredictable prior to experimentation. Finally, the ability to create sophisticated virtual patients may be of use in informing power calculations for real-world RCTs. Because the number of agents may be arbitrarily controlled, it would be possible to run several simulated RCTs using the best-available patient models, in order to assure that the statistical power of a planned RCT will be sufficient to detect likely results. Similarly, because effect-sizes of treatment may be subjected to sensitivity analysis, agent-based simulations may be useful in determining the power needed to detect the minimal effect size in a realistic simulated population.

## Limitations, Future Work, and Conclusions

### Limitations

The real-world retrospective cohort for this model is relatively small, representing only 535 patients. We believe that with a larger sample size, a greater number of statistically significant predictors could be found. Additionally, while the recent HbA1c of the patients was not found to be a significant predictor of DR state, we believe that a longer term measure will significantly inform the model, based on known relationships between HbA1c and DR progression [19]. The seemingly protective action of BMI on the multiple regression model is possibly due to the fact that our data is truncated on the low end; that is, our sample includes very few patients of normal weight, or underweight. However, it is also possible that our population is not well measured by BMI in terms of adiposity compared with lean muscle mass. Because this effect was an artifact found in the real-world data, it would be present in any predictive model based on the same real-world cohort.

We were not able to find statistically significant predictive value for the various stages of NPDR (mild, moderate, severe), a result we believe to be due to the small sample size and ‘snapshot’ nature of the data abstraction. Therefore, for the purposes of this pilot study, it was decided to restrict prediction of DR progression to NPDR versus PDR, for which we have good significance indicators. We feel this is an appropriate restriction for two reasons. First, our primary aim is to develop a cohort which will allow us to predict the demand for pan-retinal photocoagulation, a laser treatment for DR which is indicated for PDR, but ordinarily not indicated for NPDR [20]. Second, it is common practice in the literature to distinguish at the level of NPDR v PDR [3].

### Future Work

The next phase of this project is to test the consequences of annual vs. bi-annual (and longer) retinopathy screening procedures. In this way we will determine the influence of mean screening interval on the rate of incident vision loss in the population. This work will involve allowing the agents to interact by competing for resources at a simulated ophthalmology clinic. This represents an application of agent-based modeling to the generation of evidence-based performance measure, in a circumstance where real-world experimentation cannot be responsibly conducted, should bi-annual or longer screening turn out to have serious consequences for patient vision. Our goal is to provide a recommendation for the appropriate screening interval to maximize access to care for all patients while preserving sighted life-years.

### Conclusions

Agent-based modeling provides a platform for the development of sophisticated simulated cohort models. These models may provide valuable insight for implementation science, translational medicine, and health economic models. Specifically, the use of agent-based modeling to examine the population of diabetic Veterans seeking care at a regional VA Medical Center shows potential for aiding in evidence-based policy and performance measure.
